# Steep Differences in Wingless Signaling Trigger Myc-Independent Competitive Cell Interactions

**DOI:** 10.1016/j.devcel.2011.06.021

**Published:** 2011-08-16

**Authors:** Jean-Paul Vincent, Golnar Kolahgar, Maria Gagliardi, Eugenia Piddini

**Affiliations:** 1 MRC National Institute for Medical Research, Mill Hill, London NW7 1AA, UK; 2 The Wellcome Trust/CRUK Gurdon Institute, University of Cambridge, Tennis Court Road, Cambridge CB2 1QN, UK

## Abstract

Wnt signaling is a key regulator of development that is often associated with cancer. Wingless, a *Drosophila* Wnt homolog, has been reported to be a survival factor in wing imaginal discs. However, we found that prospective wing cells survive in the absence of Wingless as long as they are not surrounded by Wingless-responding cells. Moreover, local autonomous overactivation of Wg signaling (as a result of a mutation in *APC* or *axin*) leads to the elimination of surrounding normal cells. Therefore, relative differences in Wingless signaling lead to competitive cell interactions. This process does not involve Myc, a well-established cell competition factor. It does, however, require Notum, a conserved secreted feedback inhibitor of Wnt signaling. We suggest that Notum could amplify local differences in Wingless signaling, thus serving as an early trigger of Wg signaling-dependent competition.

## Graphical abstract

**Graphical Abstract:**

**Figure:**
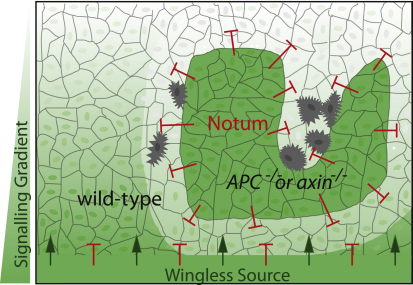

**Highlights:**

► Wingless-blind cells survive within large groups but die as part of small clones ► Patches of *APC* or *axin* mutant cells trigger apoptosis in surrounding normal cells ► The competitive nature of *axin* mutant cells is not mediated by Myc ► Tissue takeover by *axin* cells requires Notum, a secreted inhibitor of Wg signaling

## Introduction

Wnts are secreted glycolipoproteins that regulate many aspects of embryonic development, including tissue patterning, cell proliferation, differentiation, and migration. Wnt signaling is also important during adult life, as it maintains a pool of undifferentiated stem cells and promotes their proliferation (Nusse et al., 2008). Moreover, deregulation of this pathway leads to uncontrolled proliferation and is associated with many types of cancers (Clevers, 2006; Segditsas and Tomlinson, 2006; Fodde and Brabletz, 2007). Wnt signaling has been extensively studied in the fruit fly, where Wingless (Wg, a founding member of the Wnt family) also regulates many developmental processes. For example, Wg signaling modulates both cell fate decisions and growth in wing imaginal discs. Since patches of wing imaginal cells that are deficient in Wg signal transduction are progressively eliminated by apoptosis, Wg signaling has also been suggested to act as a survival factor in this tissue (Giraldez and Cohen, 2003; Johnston and Sanders, 2003).

Here we report that Wg signaling is not intrinsically required for wing cell survival: wing cells that are made unable to respond to Wg do survive, as long as they are surrounded by other nonresponsive or growth-compromised cells. This highlights the importance of relative, as opposed to absolute, levels of signaling. Indeed, normal cells are eliminated if they are surrounded by cells that overactivate Wg signal transduction (e.g., as a result of a mutation in *axin* or *APC*). Further characterization of the process allows us to conclude that it does not require modulation of Myc activity and that it requires the secreted negative feedback inhibitor encoded by *notum*.

## Results and Discussion

### Survival of Wg-Insensitive Cells Depends on the Fitness of Surrounding Cells

It is well established that, in *Drosophila* wing imaginal discs, small patches of cells that cannot transduce the Wg signal (e.g., lacking the two Wg receptors Fz and Fz2) are eliminated by apoptosis (Giraldez and Cohen, 2003; Johnston and Sanders, 2003). However, we found that posterior (P) compartments made entirely of *wg* or *fz fz2* mutant cells survive to the end of larval life. Although these compartments are reduced in size, they show no significant increase in the rate of apoptosis (see Figures S1A–S1E″ available online; see also Couso et al., 1994; Piddini and Vincent, 2009; Baena-Lopez et al., 2009). Therefore, Wg is not an absolute survival factor in wing imaginal discs. Instead, it appears that cell survival/death is influenced by Wg signaling in reference to the extent of signaling in surrounding cells.

**Figure 1:**
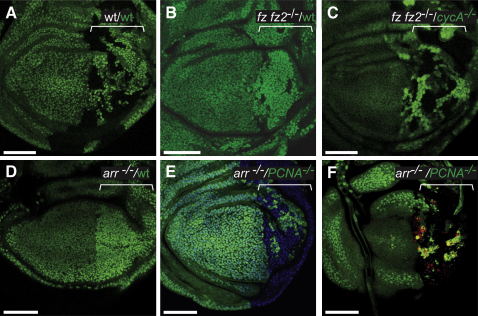
Survival of Wg-Deficient Cells Depends on the Fitness of Surrounding Cells Twin clones (marked with 2XGFP or the absence of GFP) were generated at maximal frequency in the posterior (P) compartment (marked by a bracket) with *engrailed-*Gal4*, UAS-FLP*. On each panel, green lettering describes the GFP-positive and white lettering the GFP-negative tissue. (A) Control disc where both twins are wild-type for Wg signaling (wt). (B) Homozygous *fz fz2* mutant cells (GFP-negative) surrounded by wild-type (2XGFP-positive) cells (n = 22/26 discs). (C) *fz fz2* (GFP-negative) cells are partially rescued when juxtaposed to growth-compromised *cycA* mutant (2XGFP-positive) neighbors (n = 11/15 discs). (D–F) Twin clones generated using *hedgehog-*Gal4*, UAS-FLP*. (D) Few or no *arr* mutant (GFP-negative) cells surrounded by wild-type (2XGFP) twin cells survive to late larval stages. (E) *arr* (GFP-negative) cells are partially rescued when juxtaposed to growth-compromised *PCNA* mutant (GFP-positive) neighbors (n = 24/24 discs). (F) A basal section from the same disc in (E), processed with antiactivated Caspase- 3 antibody to show apoptotic cells. Most of the Caspase-3 immunoreactivity is in the *PCNA^−/−^* territory. All panels show single confocal sections. For all figures scale bars represent 50 μm, Anterior is left and Dorsal on top. Detailed genotypes are listed in the Experimental Procedures.

To further characterize the influence of surrounding cells on the survival of Wg signaling-deficient cells, mosaic tissues containing equal numbers of distinctly marked progenitor cells were generated. In control experiments, *engrailed-*Gal4-driven *UAS-Flp* was used to generate two populations of wild-type cells, one marked by the absence of GFP and the other carrying two copies of a GFP-producing transgene (2xGFP). As can be seen in Figure 1A, these two cell populations came to occupy approximately equal surface areas. For genetically distinct populations (e.g., mutant and wild-type at a given locus), any departure from parity would indicate differential rates of growth or apoptosis. This was indeed the case when the same FRTs and source of Flp were used to create mosaic imaginal discs containing a mixture of *fz fz2* mutant cells (GFP-negative) and wild-type cells (2xGFP). Few *fz fz2* GFP-negative mutant cells were recovered in such P compartments (compare the extent of the GFP-negative domains in Figures 1A and 1B). This result confirms that, in agreement with previous observations (Giraldez and Cohen, 2003; Johnston and Sanders, 2003), Wg-unresponsive cells fail to thrive when they grow alongside wild-type cells. However when the same recombination system was used to create mosaics of *fz fz2* (GFP-negative) and *cyclinA* (*cycA*) mutant (2xGFP) cells, which are proliferation impaired (Figure S1F) (Lehner and O'Farrell, 1989), a different result was obtained. This time *fz fz2* mutant cells colonized the P compartment similarly to wild-type cells in control mosaics (compare the GFP-negative territories in Figures 1A and 1C).

To confirm and extend the above conclusion, we assessed the behavior of cells lacking *arrow*, which encodes the *Drosophila* homolog of LRP5/6, an essential Wnt coreceptor (Wehrli et al., 2000). Using *hedgehog-*Gal4 and *UAS-Flp,* we generated a large number of *arrow* mutant cells (GFP-negative) during the first instar stage. In all cases, *arrow* mutant cells contributed very little (data not shown; 4/8 discs) or no (Figure 1D; 4/8 discs) tissue to third instar discs. We could be sure that *arrow* mutant cells had been generated (and subsequently eliminated) because the remainder of the P compartment was made of 2xGFP cells, which could only arise by the same recombination event that produced *arrow* mutant cells. It is likely that in these imaginal discs, *arrow* mutant cells are eliminated by apoptosis (Giraldez and Cohen, 2003; Johnston and Sanders, 2003). In principle, apoptosis could be the primary event or it could be an indirect consequence of delamination from the epithelium (Widmann and Dahmann, 2009). This can be difficult to assess rigorously, as apoptosis leads to delamination. Nevertheless, in heat-shock-induced *arrow* mutant patches, some caspase-positive *arrow* mutant cells were detected on the apical side of the epithelium before any sign of delamination, which normally occurs on the basal side (Figure S1G). Therefore, it is likely that apoptosis contributes directly to the elimination of *arrow* mutant cells, perhaps in combination with delamination. If, instead of being surrounded by wild-type cells, *arrow* mutant (GFP-negative) cells were interspersed with *PCNA* mutant (2xGFP) cells, which divide only a few times before exhaustion of perduring protein (Henderson et al., 1994; Tsuda et al., 2006), their contribution to imaginal discs was strongly enhanced (see the extent of the GFP-negative territory in Figure 1E). Importantly, this increased contribution to disc tissue was correlated with a low rate of apoptosis (Figure 1F). In *arrow^−/−^/PCNA^−/−^* mosaic compartments, the surface density of *arrow^−/−^* Caspase-3-positive cells was only 12.26 per unit area ± 6.8 (unit area = 10^4^ μm^2^). This is in marked contrast with *arrow^−/−^/*wild-type mosaics (as shown in Figure S1G) where the surface density of *arrow^−/−^* caspase-positive cells was found to be 52.8 per unit area ± 20.3. Altogether these results show that cells unable to respond to Wingless can survive and contribute to imaginal discs if they are surrounded by other signaling-deficient or by growth-compromised cells.

### Cells that Autonomously Overactivate Wg Signaling Kill Their Wild-Type Neighbors

**Figure 2:**
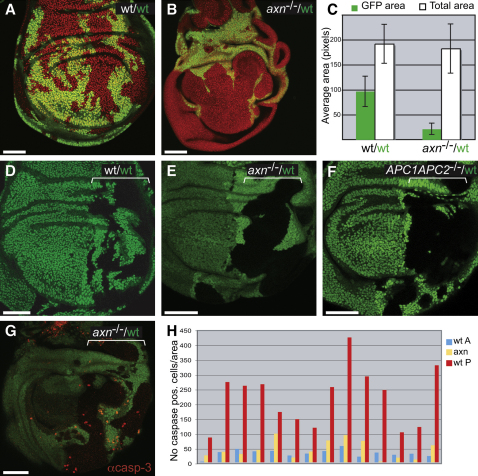
Cells that Autonomously Overactivate Wg Signaling Outcompete Their Wild-Type Neighbors (A and B) Twin clones induced with *escargot-*Gal4*, UAS-FLP*. (A) Wild-type control clones. (B) *axin* mutant (GFP-negative) and wild-type (GFP-positive) twin clones. (C) Average surface area (±SD) of the pouch and of the GFP-positive domain in 14 control discs (genotype as in A) and 20 experimental discs (as in B). The area of the GFP domain (wild-type) is significantly different in the two conditions (p = 7E-12). (D) Control, wild-type twins generated throughout the P compartment (bracket). (E) As in (B), *axin* mutant cells affect wild-type (2XGFP) neighbor twins (compare to wild-type 2XGFP cells in control disc in D). (F) *APC* mutant (GFP-negative) and wild-type (2XGFP) twin clones. (G) Mid-third instar disc of the same genotype as in (E) stained with antiactivated Caspase-3 (red). An optical section through the basal region of the epithelium (where apoptotic cells accumulate) is shown. (H) Quantification of the number of caspase-positive cells per surface area in 14 wing discs of the same genotype as in (G). For each disc, caspase-positive cells were counted throughout the thickness of the wing pouch in three different areas (control A cells in blue, wild-type P cells in red, and *axin* mutant cells in yellow). Blue and red bars are significantly different p = 3.5E-6). In all panels, micrographs are single sections and GFP is shown in green. DAPI is in red in (A) and (B).

The above results suggest that a crucial parameter in cell survival decisions is the relative level of Wg signaling activity in neighboring cells. To further test this possibility, we turned to situations of local excess signaling. Mutations in either *axin* or *APC*, two tumor suppressor genes encoding negative regulators of Wg signaling, cause cell autonomous overactivation of the Wg pathway (Ikeda et al., 1998; Akong et al., 2002). Such mutations have previously been shown to trigger overproliferation in wing discs (Giraldez and Cohen, 2003; Hayward et al., 2006) and a variety of mammalian tissues (Segditsas and Tomlinson, 2006; Liu et al., 2000; Satoh et al., 2000; Webster et al., 2000). Mutant clones were generated and the effect on the growth of surrounding wild-type (GFP-positive) tissue was assessed. In one set of experiments, *axin* mutant clones were induced using *UAS-Flp* and *esgargot-*Gal4, which is expressed throughout the disc from the first instar stage. As before, control GFP-negative and 2xGFP cells (both otherwise wild-type and therefore of equal fitness) were first generated to assess the extent of recombination induced by this source of Flp. Each cell population colonized approximately half of the disc, as expected (Figure 2A). If, however, wild-type (GFP-positive) cells were confronted with *axin* mutant cells during disc development, they became significantly underrepresented at the third instar (Figure 2B; compare the extent of wild-type GFP-positive territory in A and B). For 14 control discs (as in A) and 20 experimental discs (as in B), we measured the size of the pouch, the central region of the disc that normally gives rise to the wing proper, as well as the area of the pouch occupied by GFP-positive cells. As can be seen in Figure 2C, while the total pouch area is roughly similar in the two conditions, the area occupied by wild-type GFP-positive cells is greatly reduced by the presence of *axin* mutant cells. Importantly, the genotype of GFP-positive cells is identical in both experimental situations and it is the presence or absence of *axin* mutant cells that affects the growth or survival of wild-type cells.

One additional feature became apparent when *axin* mutant cells (GFP-negative) were generated only in the P compartment (using *engrailed-*Gal4 and *UAS-Flp,* Figure 2E) or only in the anterior (A) compartment (using *Ci-*Gal4 and *UAS-Flp,* Figure S2A). As before, the progeny of such cells were overrepresented at the expense of wild-type 2XGFP cells (Figure 2E and Figure S2A, compared with control mosaics in Figure 2D). However, *axin* mutant clones generated in one compartment did not spill across the compartment boundary. Importantly, the presence of *axin* mutant patches did not affect larval growth, and therefore the underrepresentation of wild-type cells was not due to shortening of the growth period (data not shown). To further confirm the above result with another mutation that boosts Wg signaling, we generated mosaic P compartments containing *APC* mutant (*APC1-APC2* double mutant) and wild-type cells. Like *axin* mutant cells, *APC* mutant cells colonized the majority of the P compartment at the expense of wild-type P cells but did not affect the wild-type A compartment (Figure 2F). We conclude that cell-autonomous activation of Wg signaling allows cells to take over prospective tissue at the expense of normal cells and that this behavior is limited by the compartment boundary.

As shown previously by others, small clones of *fz fz2* or *arrow* mutant cells undergo apoptosis (Giraldez and Cohen, 2003; Johnston and Sanders, 2003) and, as described above, this depends on the nearby presence of wild-type, signal-transducing cells (Figure 1 and Figure S1). To test if autonomous overactivation of Wg signaling triggers apoptosis in normal surrounding cells, wild-type/*axin*^−/−^ mosaic discs were stained with antiactivated Caspase-3 (Figure 2G). Relatively young discs (mid-third instar) were analyzed so that dying cells could be visualized before their elimination. Since mutant clones were generated only in the P compartment (using *engrailed-*Gal4, *UAS-Flp*), activated Caspase-3 immunoreactivity in the anterior (A) compartment provided an internal reference for the background level of apoptosis. Only occasional caspase-positive cells were detected in this compartment, as expected from previous reports that relatively little apoptosis takes place in wild-type imaginal discs (Giraldez and Cohen, 2003). By contrast, many caspase-positive cells were seen in wild-type posterior cells. For 14 discs, the density of caspase-positive cells was measured in the control A compartment, in posterior wild-type cells, and in posterior *axin* mutant cells (Figure 2H). Caspase-positive immunoreactivity was consistently higher in wild-type posterior cells than in control anterior cells (compare red and blue bars in H; p = 3.5E-6). Therefore, *axin* mutant cells trigger increased apoptosis in neighboring wild-type cells. Such nonautonomous induction of apoptosis, together with a cell-autonomous increase in growth (see Figure S2B and Martín et al., 2009), is likely to enable *axin* mutant cells to overcolonize the tissue. Altogether these results indicate that relative differences in Wg signaling activity lead to competitive cell interactions that determine whether cells die or survive.

### The Competitive Nature of *axin* Mutant Cells Is Myc Independent

The effect of local differences in Wg signaling is reminiscent of the phenomenon of cell competition. As shown many years ago, cells experiencing reduced ribosomal activity (due to mutation in one of the *Minute* genes, which encode ribosomal subunits) are eliminated when they grow alongside wild-type cells (Morata and Ripoll, 1975; Simpson, 1979; Simpson and Morata, 1981) but are viable if surrounded by other ribosome-deficient cells. More recently, it was recognized that local differences in the level of Myc, a key regulator of ribosome biosynthesis (Grewal et al., 2005; Teleman et al., 2008) also trigger cell competition in wing discs. For example, cells with extra dosage of *myc* outcompete surrounding wild-type cells (de la Cova et al., 2004; Moreno and Basler, 2004). This may be a general phenomenon, since a relative excess of Myc has been shown to bring about cell competition among stem cells in the adult *Drosophila* ovary (Rhiner et al., 2009) and to be the underlying cause of competition induced by other genetic lesions (Froldi et al., 2010; Ziosi et al., 2010).

**Figure 3:**
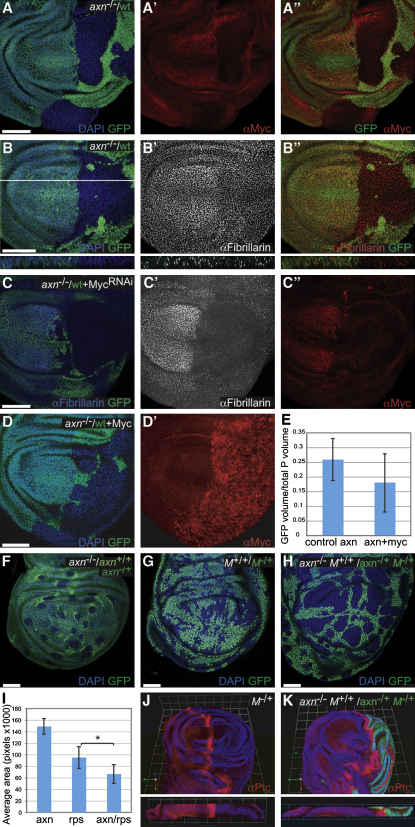
The Competitive Nature of *axin* Mutant Cells Is Myc Independent and Boosted by *Minute*-Induced Competition In all the *axin*^−/−^/wild-type mosaics, *axin* mutant cells are GFP-negative and wild-type cells are GFP-positive. (A–A″) Myc expression, as detected with an antibody. (B–B″) Nucleolar size (as judged by anti-Fibrillarin staining), shown here in a mid-third instar disc. Bottom panels show reconstruction of a cross-section at the position indicated by the white line in (B). (C–C″) Uniform downregulation of *myc* by RNAi throughout the P compartment (with *engrailed*-Gal4). (C). The efficacy of the RNAi is demonstrated by the downregulation of Fibrillarin (C') and Myc protein levels (C″). (D and D′) Uniform Myc overexpression throughout the P compartment (with *engrailed*-Gal4, anti-Myc shown in D'). (E) Histogram showing the average fraction of the P compartment occupied by GFP-positive tissue in control discs (genotype as in A or B, n = 8) and in Myc overexpressing discs (genotype as in D, n = 14). Staining and genotypes are indicated on each panel. (F–H) Clones induced simultaneously (using *hs-flp*) and then allowed to grow for 58 hr before fixation at the end of larval life (see Experimental Procedures). The genotype of GFP-negative cells (“winners”) is indicated in white. (I) Histogram showing the average size (±SD) of the GFP-positive area within the pouch in discs of the same genotype as in (F) (left; n = 4), (G) (middle; n = 8), or (H) (right; n = 4). Asterisk indicates statistical significance; p = 0.027. (J and K) 3D reconstruction of wing discs stained with anti-Ptc (red) to mark the A cells that line the A/P boundary (nonboundary A cells express Ptc weakly). Bottom panels show a cross-section (apical facing up). (J) shows a control disc (*Minute^−/+^*) while K shows a wing disc with the A compartment entirely made of GFP-negative, dual competitor cells (genotype as indicated). Note the shift of the Ptc stripe and the reduced size of the P compartment (*axin^−/+^ Minute^−/+^*; GFP-positive). In all panels, GFP is shown in green and DAPI in blue. (C), (D), and (F)–(H) show single sections while (A)–(A ″), (B–B″), (C′ and C″) and (D′) show projections.

Given the documented involvement of Myc in cell competition, increased Myc activity might conceivably account for the competitive nature of *axin* mutant cells. However, staining with anti-Myc showed that the level of Myc protein is reduced in *axin* and *APC* mutant cells (Figures 3A–A″ and Figure S3; see also Duman-Scheel et al., 2004). As a further test, we used anti-Fibrillarin, a marker of nucleolar size, which is positively regulated by Myc (Grewal et al., 2005). No increased staining could be detected within the *axin* mutant cells (Figures 3B–B”). These observations suggest that the competitive advantage of *axin* cells is not mediated by increased Myc level or activity. To further assess the possible involvement of Myc, we tested whether reduction or increase in Myc expression had any effect on the competitive nature of *axin* mutant cells. As expected from the essential role of Myc in cell growth, expression of a highly active *myc* RNAi transgene led to a severe impairment in wing disc growth, even in discs harboring *axin* mutant clones (data not shown). However, milder RNAi-mediated knockdown of *myc* (with a weaker RNAi transgene) throughout the P compartment (functionally confirmed with anti-Myc and anti-Fibrillarin; Figures 3C′ and C″) had no noticeable effect on the ability of *axin* mutant cells to take over this compartment (Figure 3C). Conversely, overexpression of Myc (using a UAS-*myc* transgene, Figure 3D′) did not enable wild-type cells to withstand the competition from *axin* mutant cells (Figures 3D and 3E).

### *axin-* and *Minute*-Induced Competitive Interactions Are Additive

The results above suggest that the competitive advantage of *axin* cells is not mediated by an increase in ribosomal number since Myc, a key regulator of ribosome production, is not involved. We therefore sought to test if a relative increase in ribosomes (e.g., by manipulating *Minute* dosage) might boost the competitive nature of *axin* mutant cells. A heat-shock-controlled Flp-expressing transgene was used to induce late (during the second instar) and sporadic recombination events. This way, and by contrast to the experiments with *engrailed-*Gal4 and *UAS-Flp*, competition was allowed to take place only during a relatively short time, thus limiting the extent of growth and cell killing and allowing any additive effect on competition to be detected. Under these conditions, *axin* (GFP-negative) mutant cells did not have enough time for extensive takeover and many wild-type (GFP-positive) cells still remained at the time of fixation (Figure 3F). Likewise, in *Minute* mosaics created with the same recombination protocol, *Minute^−/+^* (GFP-positive) cells still contributed a substantial amount of pouch tissue when put in competition with GFP-negative *Minute^+/+^* cells (Figure 3G). Importantly, in a situation of dual competition (GFP-negative *axin^−/−^ M^+/+^* versus GFP-positive *axin^−/+^ M^−/+^*), the GFP-positive cells were outcompeted in a more pronounced way (Figure 3H). Quantification of these results is shown in Figure 3I, where it is apparent that the two forms of competition are additive. This suggests that the competitive advantage of *axin* mutant cells is increased if they are better endowed with ribosomes than their neighbors. This was confirmed by studying the behavior of anterior (A) compartments made entirely of dually competitive cells. We used *ci-*Gal4 and *UAS-Flp* in a *Minute^−/+^* background to generate discs with the A compartment made entirely of *axin^−/−^ M^+/+^* (GFP-negative) cells, while the P compartment was composed of *axin^−/+^ M^−/+^* (GFP-positive) cells. In this situation, A cells appeared to push the A-P compartment boundary (labeled by the expression of Ptc) toward the posterior, thus allowing only a small P compartment to develop (Figure 3K, compare to control disc in 3J). Such dramatic behavior is not seen for *axin* mutant cells alone (Figures 2E and 2F). Moreover, *Minute* competition largely respects the compartment boundary (Simpson, 1979; Simpson and Morata, 1981), except for one particularly strong *Minute* mutation (Brower et al., 1981). Therefore, we conclude that the competitive behavior of *axin* mutant cells is boosted if they acquire a relative advantage in translational potential and that this allows the effect of cell competition to overcome the restriction imposed by the compartment boundary.

### The Competitive Advantage of *axin* Cells Requires Notum Activity

**Figure 4:**
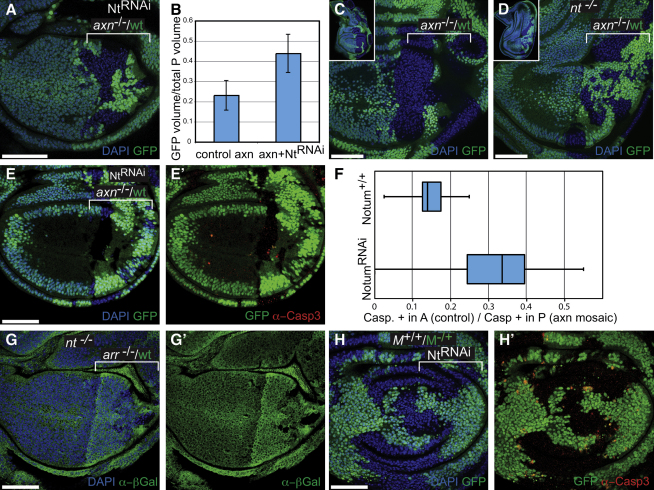
*notum* Mediates Wnt-Induced but Not *Minute* Competition (A-E) Twin patches of *axin* mutant (GFP-negative) and *axin+* (GFP-positive) cells. (A) *notum* knockdown throughout the P compartment (with *engrailed*-Gal4). (B) The average fractional volume (±SD) of the P compartment occupied by GFP-positive tissue in control discs (*axin*^−/−^/wild-type mosaics; n = 6; as in Figure 2E) is less than in discs that, in addition, express the *notum* RNAi transgene (n = 8; genotype as in A); p = 0.00058. (C and D) In *notum* mutant larvae (D, genotype *wf ^141^*/*Df3(3L)st-f13*), *axin^+^* cells (GFP-positive) are no longer outcompeted by *axin* mutant cells (n = 19/25; compare to competition in a *notum^+/+^* background as in (C), where competition was observed in 15/18 cases). Top left insets show the imaginal discs in their entirety. (E and E′) Experiments as in (A), stained for activated Caspase-3. (F) For individual discs, the surface density of caspase-positive cells in A (control) and P (outcompeted) cells was measured to generate an A/P ratio (the lower the ratio the stronger cell competition). Such ratios were compared for discs with normal (*notum^+/+^*; 14 discs) or knocked down *notum* activity (*notum* RNAi; 15 discs). (G and G′) Twin clones of *arrow* mutant (β-Gal-negative) and wild-type (β-Gal-positive) cells in a *notum* mutant background (compare with Figures 1D and 1E). (H and H′) Knockdown of *notum* by RNAi throughout the P compartment with *engrailed*-Gal4 does not affect *Minute^−/+^* (GFP-positive) versus wild-type (GFP-negative) cell competition. Active Caspase-3 staining in red.

Since one would expect competition to be mediated by intercellular communication, it seems likely that autonomous activation of Wg signaling triggers the release of secondary signals that induce the elimination of surrounding wild-type cells (see, e.g., Senoo-Matsuda and Johnston, 2007 and Rhiner et al., 2010). One candidate contributory signal is the secreted glypican-specific phospholipase encoded by *notum*, also known as *wingful* (Gerlitz and Basler, 2002; Giráldez et al., 2002). Activation of Wg signaling triggers the expression of Notum, which, in turn, suppresses the response to Wg in surrounding cells (Piddini and Vincent, 2009), possibly by cleaving glypicans from the surface of cells (Kreuger et al., 2004; Traister et al., 2007). We tested whether Notum participates in Wg signaling-dependent competition by knocking down *notum* in *axin^−/−^*/wild-type mosaic tissue. *engrailed-*Gal4 was used both to induce *axin* mutant patches in the P compartment (as before), and to drive expression of a hairpin RNAi construct against *notum* (along with UAS-Dicer to boost silencing). In such imaginal discs, wild-type GFP-positive cells contributed significantly more tissue to the pouch than in control discs where *notum* had not been knocked down (compare Figure 4A to Figure 2E and see quantification in Figure 4B). This was accompanied by a reduction in the contribution of *axin* cells to the pouch, whose size was largely unaffected. Similar results were obtained when *notum* activity was reduced by mutation, as indicated by the behavior of *axin^−/−^*/wild-type mosaics in a heteroallelic *notum* mutant background (compare Figures 4C and 4D). Therefore, loss of *notum* blunts the ability of *axin* mutant cells to take over the wing pouch. Importantly, this is correlated with a significant improvement in the survival rate of axin^+/+^ cells surrounding axin^−/−^ cells, as assayed with anti-Caspase-3 staining (compare Figure 2G and Figures 4E–4E′ and see quantitative analysis in Figure 4F). We conclude that Notum is required for *axin* mutant cells to trigger apoptosis in surrounding normal cells and hence to gain a competitive advantage.

On the basis of the expected epistatic relationship between *notum* on one hand and *arrow* or *fz fz2* on the other hand (*notum* is expected to be upstream), it seems unlikely that Notum would contribute to the elimination of cells lacking these signaling receptors. However, in *notum* mutant discs (same heteroallelic combination as above), the recovery of *arrow* mutant clones was moderately improved (Figures 4G and 4G′, compare to Figure 1D; n = 7/10). It is conceivable that this is mediated by an effect on other signaling pathways, such as those activated by Hedgehog and Decapentaplegic, which also are modulated by glypicans (Fujise et al., 2003; Akiyama et al., 2008; Ayers et al., 2010). Thus, removal of Notum may increase the overall level of growth factor signaling in *arrow* mutant cells, resulting in a detectable cytoprotective effect. Nevertheless, we found that *notum* does not contribute to *Minute* competition since apoptosis was induced at *Minute*^−*/+*^/*Minute^+/+^* interfaces irrespectively of whether *notum* was knocked down or not (compare the A and P compartments in Figures 4H and 4H′). Altogether these results identify Notum as a soluble extracellular mediator of Wnt-induced competition, but not of *Minute* competition.

### Conclusion

One conclusion of our work is that Wg signaling is not intrinsically required for wing cell survival and that, instead, competitive cell interactions triggered by local differences in Wingless signal transduction influence survival decisions. Such local differences can arise between clones that either cannot transduce the signal (e.g., *fz fz2* or *arrow* mutant) or overactivate signaling (e.g., *axin* or *APC* mutant). In both cases, the low signaling cells are eliminated. It has been suggested that other forms of cell competition (Baker and Li, 2008; Moreno, 2008) could be relevant to cancer. Moreover, mutations in *axin* and *APC* are found in a variety of cancers (Clevers, 2006; Segditsas and Tomlinson, 2006; Fodde and Brabletz, 2007). Therefore, it is conceivable that humans precancerous *APC* or *axin* mutant cells could acquire a competitive advantage that enables them to clear surrounding normal tissue, thus contributing to tissue colonization. As we have shown, this is not mediated by local differences in the activity of Myc, a key regulator of ribosomal activity and a well-established factor of cell competition (de la Cova et al., 2004; Moreno and Basler, 2004). In fact, the competitive nature of *axin* mutant cells was boosted by experimentally increasing their relative content of functional ribosomes. By analogy, in humans, loss of *axin* (or *APC*) and increased translational potential are two features that could have additive effects in boosting early tumor progression and enabling tumors to overcome preexisting barriers to tissue growth.

Although the cell biological basis of Wg signaling-induced competition remains to be elucidated, we have identified one important mediator, the secreted phospholipase encoded by *notum*. As we have shown, *notum* knockdown prevents *axin* mutant cells from taking over the wing pouch even though these cells are themselves insensitive to Notum activity (Piddini and Vincent, 2009). Therefore, the overgrowth of *axin* mutant cells is not solely an autonomous consequence of overactive Wg signaling. As a result of high signaling activity, *axin* mutant cells secrete Notum, which inhibits signaling in neighboring wild-type cells (Figure S4). Thus, an initial signaling difference is amplified and then transduced into downstream events that lead to the elimination of normal cells, which is required for axin mutant cells to overgrow and take over the tissue.

## Experimental Procedures

### *Drosophila* Stocks

Detailed information about the *Drosophila* stocks is given in the Supplemental Experimental Procedures along with a list of all the genotypes analyzed.

### Antibody Staining

The following primary antibodies were used: mouse anti-Wg 4D4 (prepared from cells obtained from the DSHB), rabbit antiactivated Caspase-3 (Cell Signaling Technology), guinea pig, anti-D-myc (a gift of M. Milan and G. Morata); mouse anti-Ptc (DSHB); rabbit anti-Phospho-Histone H3 (Upstate Biotechnology); mouse anti-Fibrillarin (AbCam); chicken anti-β-Gal (AbCam). Secondary antibodies used were Alexa-conjugated anti-mouse, anti-rabbit, and anti-guinea pig (Molecular Probes, Eugene).

### Heat-Shock Induction of Mutant Clones

Mitotic recombination was induced by heat-shocking larvae for 1 hr at 37°C. To compare the strength of competition between *axin*, *Minute*, and *axin*+*Minute* (Figures 4A–4D), larvae of the relevant genotypes were heat-shocked simultaneously at various stages of development and then allowed to grow for the same length of time. Those reaching the end of larval life (anterior spiracle eversion) simultaneously (58 ± 1 hr in the experiment shown) were fixed and analyzed. This ensured that competition was allowed to happen over the same time across different larvae and genotypes even if they grew at different rates (M^−/+^ larvae are slower than normal).

### Imaging, Image Analysis, and Quantifications

Details on image analysis, tissue volume measurements and counting of Caspase-3-positive cells are provided in Supplemental Experimental Procedures. All error bars and error estimates represent one standard deviation. Statistical significance was assessed using Student's paired t tests.

## Supplemental Information

Document S1. Four Figures and Supplemental Experimental Procedures

## References

[bib1] Akiyama T., Kamimura K., Firkus C., Takeo S., Shimmi O., Nakato H. (2008). Dally regulates Dpp morphogen gradient formation by stabilizing Dpp on the cell surface. Dev. Biol..

[bib2] Akong K., Grevengoed E.E., Price M.H., McCartney B.M., Hayden M.A., DeNofrio J.C., Peifer M. (2002). Drosophila APC2 and APC1 play overlapping roles in wingless signaling in the embryo and imaginal discs. Dev. Biol..

[bib3] Ayers K.L., Gallet A., Staccini-Lavenant L., Thérond P.P. (2010). The long-range activity of Hedgehog is regulated in the apical extracellular space by the glypican Dally and the hydrolase Notum. Dev. Cell.

[bib4] Baena-Lopez L.A., Franch-Marro X., Vincent J.P. (2009). Wingless promotes proliferative growth in a gradient-independent manner. Sci. Signal..

[bib5] Baker N.E., Li W. (2008). Cell competition and its possible relation to cancer. Cancer Res..

[bib6] Brower D.L., Lawrence P.A., Wilcox M. (1981). Clonal analysis of the undifferentiated wing disk of Drosophila. Dev. Biol..

[bib7] Clevers H. (2006). Wnt/beta-catenin signaling in development and disease. Cell.

[bib8] Couso J.P., Bishop S.A., Martinez Arias A. (1994). The wingless signalling pathway and the patterning of the wing margin in Drosophila. Development.

[bib9] de la Cova C., Abril M., Bellosta P., Gallant P., Johnston L.A. (2004). Drosophila myc regulates organ size by inducing cell competition. Cell.

[bib10] Duman-Scheel M., Johnston L.A., Du W. (2004). Repression of dMyc expression by Wingless promotes Rbf-induced G1 arrest in the presumptive Drosophila wing margin. Proc. Natl. Acad. Sci. USA.

[bib11] Fodde R., Brabletz T. (2007). Wnt/beta-catenin signaling in cancer stemness and malignant behavior. Curr. Opin. Cell Biol..

[bib12] Froldi F., Ziosi M., Garoia F., Pession A., Grzeschik N.A., Bellosta P., Strand D., Richardson H.E., Pession A., Grifoni D. (2010). The lethal giant larvae tumour suppressor mutation requires dMyc oncoprotein to promote clonal malignancy. BMC Biol..

[bib13] Fujise M., Takeo S., Kamimura K., Matsuo T., Aigaki T., Izumi S., Nakato H. (2003). Dally regulates Dpp morphogen gradient formation in the Drosophila wing. Development.

[bib14] Gerlitz O., Basler K. (2002). Wingful, an extracellular feedback inhibitor of Wingless. Genes Dev..

[bib15] Giraldez A.J., Cohen S.M. (2003). Wingless and Notch signaling provide cell survival cues and control cell proliferation during wing development. Development.

[bib16] Giráldez A.J., Copley R.R., Cohen S.M. (2002). HSPG modification by the secreted enzyme Notum shapes the Wingless morphogen gradient. Dev. Cell.

[bib17] Grewal S.S., Li L., Orian A., Eisenman R.N., Edgar B.A. (2005). Myc-dependent regulation of ribosomal RNA synthesis during Drosophila development. Nat. Cell Biol..

[bib18] Hayward P., Balayo T., Martinez Arias A. (2006). Notch synergizes with axin to regulate the activity of armadillo in Drosophila. Dev. Dyn..

[bib19] Henderson D.S., Banga S.S., Grigliatti T.A., Boyd J.B. (1994). Mutagen sensitivity and suppression of position-effect variegation result from mutations in mus209, the Drosophila gene encoding PCNA. EMBO J..

[bib20] Ikeda S., Kishida S., Yamamoto H., Murai H., Koyama S., Kikuchi A. (1998). Axin, a negative regulator of the Wnt signaling pathway, forms a complex with GSK-3beta and beta-catenin and promotes GSK-3beta-dependent phosphorylation of beta-catenin. EMBO J..

[bib21] Johnston L.A., Sanders A.L. (2003). Wingless promotes cell survival but constrains growth during Drosophila wing development. Nat. Cell Biol..

[bib22] Kreuger J., Perez L., Giraldez A.J., Cohen S.M. (2004). Opposing activities of Dally-like glypican at high and low levels of Wingless morphogen activity. Dev. Cell.

[bib23] Lehner C.F., O'Farrell P.H. (1989). Expression and function of Drosophila cyclin A during embryonic cell cycle progression. Cell.

[bib24] Liu W., Dong X., Mai M., Seelan R.S., Taniguchi K., Krishnadath K.K., Halling K.C., Cunningham J.M., Boardman L.A., Qian C. (2000). Mutations in AXIN2 cause colorectal cancer with defective mismatch repair by activating beta-catenin/TCF signalling. Nat. Genet..

[bib25] Martín F.A., Herrera S.C., Morata G. (2009). Cell competition, growth and size control in the Drosophila wing imaginal disc. Development.

[bib26] Morata G., Ripoll P. (1975). Minutes: mutants of drosophila autonomously affecting cell division rate. Dev. Biol..

[bib27] Moreno E. (2008). Is cell competition relevant to cancer?. Nat. Rev. Cancer.

[bib28] Moreno E., Basler K. (2004). dMyc transforms cells into super-competitors. Cell.

[bib29] Nusse R., Fuerer C., Ching W., Harnish K., Logan C., Zeng A., ten Berge D., Kalani Y. (2008). Wnt signaling and stem cell control. Cold Spring Harb. Symp. Quant. Biol..

[bib30] Piddini E., Vincent J.P. (2009). Interpretation of the wingless gradient requires signaling-induced self-inhibition. Cell.

[bib31] Rhiner C., Díaz B., Portela M., Poyatos J.F., Fernández-Ruiz I., López-Gay J.M., Gerlitz O., Moreno E. (2009). Persistent competition among stem cells and their daughters in the Drosophila ovary germline niche. Development.

[bib32] Rhiner C., López-Gay J.M., Soldini D., Casas-Tinto S., Martín F.A., Lombardía L., Moreno E. (2010). Flower forms an extracellular code that reveals the fitness of a cell to its neighbors in Drosophila. Dev. Cell.

[bib33] Satoh S., Daigo Y., Furukawa Y., Kato T., Miwa N., Nishiwaki T., Kawasoe T., Ishiguro H., Fujita M., Tokino T. (2000). AXIN1 mutations in hepatocellular carcinomas, and growth suppression in cancer cells by virus-mediated transfer of AXIN1. Nat. Genet..

[bib34] Segditsas S., Tomlinson I. (2006). Colorectal cancer and genetic alterations in the Wnt pathway. Oncogene.

[bib35] Senoo-Matsuda N., Johnston L.A. (2007). Soluble factors mediate competitive and cooperative interactions between cells expressing different levels of Drosophila Myc. Proc. Natl. Acad. Sci. USA.

[bib36] Simpson P. (1979). Parameters of cell competition in the compartments of the wing disc of Drosophila. Dev. Biol..

[bib37] Simpson P., Morata G. (1981). Differential mitotic rates and patterns of growth in compartments in the Drosophila wing. Dev. Biol..

[bib38] Teleman A.A., Hietakangas V., Sayadian A.C., Cohen S.M. (2008). Nutritional control of protein biosynthetic capacity by insulin via Myc in Drosophila. Cell Metab..

[bib39] Traister A., Shi W., Filmus J. (2007). Mammalian Notum induces the release of glypicans and other GPI-anchored proteins from the cell surface. Biochem. J..

[bib40] Tsuda L., Kaido M., Lim Y.M., Kato K., Aigaki T., Hayashi S. (2006). An NRSF/REST-like repressor downstream of Ebi/SMRTER/Su(H) regulates eye development in Drosophila. EMBO J..

[bib41] Webster M.T., Rozycka M., Sara E., Davis E., Smalley M., Young N., Dale T.C., Wooster R. (2000). Sequence variants of the axin gene in breast, colon, and other cancers: an analysis of mutations that interfere with GSK3 binding. Genes Chromosomes Cancer.

[bib42] Wehrli M., Dougan S.T., Caldwell K., O'Keefe L., Schwartz S., Vaizel-Ohayon D., Schejter E., Tomlinson A., DiNardo S. (2000). arrow encodes an LDL-receptor-related protein essential for Wingless signalling. Nature.

[bib43] Widmann T.J., Dahmann C. (2009). Wingless signaling and the control of cell shape in Drosophila wing imaginal discs. Dev. Biol..

[bib44] Ziosi M., Baena-López L.A., Grifoni D., Froldi F., Pession A., Garoia F., Trotta V., Bellosta P., Cavicchi S., Pession A. (2010). dMyc functions downstream of Yorkie to promote the supercompetitive behavior of hippo pathway mutant cells. PLoS Genet..

