# Supplementary material for: Steep Differences in Wingless Signaling Trigger Myc-Independent Competitive Cell Interactions
Source: Dev Cell. 2011 Aug 16;21(2):366–74. doi: 10.1016/j.devcel.2011.06.021 (PMC3209557; doi:10.1016/j.devcel.2011.06.021)
Supplement: Document S1. Four Figures and Supplemental Experimental Procedures [file mmc1.pdf]

Developmental Cell, Volume *21*

Supplemental Information

Steep Differences in Wingless Signaling Trigger Myc-Independent Competitive Cell Interactions

Jean-Paul Vincent, Golnar Kolahgar, Maria Gagliardi, and Eugenia Piddini

Inventory of Supplemental Information

Supplemental figures

Figure S1, related to Figure 1. It shows that the survival of cells lacking Wg signalling depends on the cellular context.

Figure S2, related to Figure 2. It shows that the behaviour of *axin*$^{-/-}$ cells is not compartment specific and is not inhibited by DIAP1 expression.

Figure S3, related to Figure 3. It provides further evidence that *axin*$^{-/-}$ and *APC*$^{-/-}$ cells downregulated Myc.

Figure S4, related to Figure 4. A model summarizing the role of Notum during competition between *axin*$^{-/-}$ or *APC*$^{-/-}$ and wild-type cells.

Supplemental experimental procedures

This section includes: a description of all the *Drosophila* stocks used; details on imaging, image analysis and quantification; list of the genotypes used in the main figures; list of the genotypes used in the supplemental figures

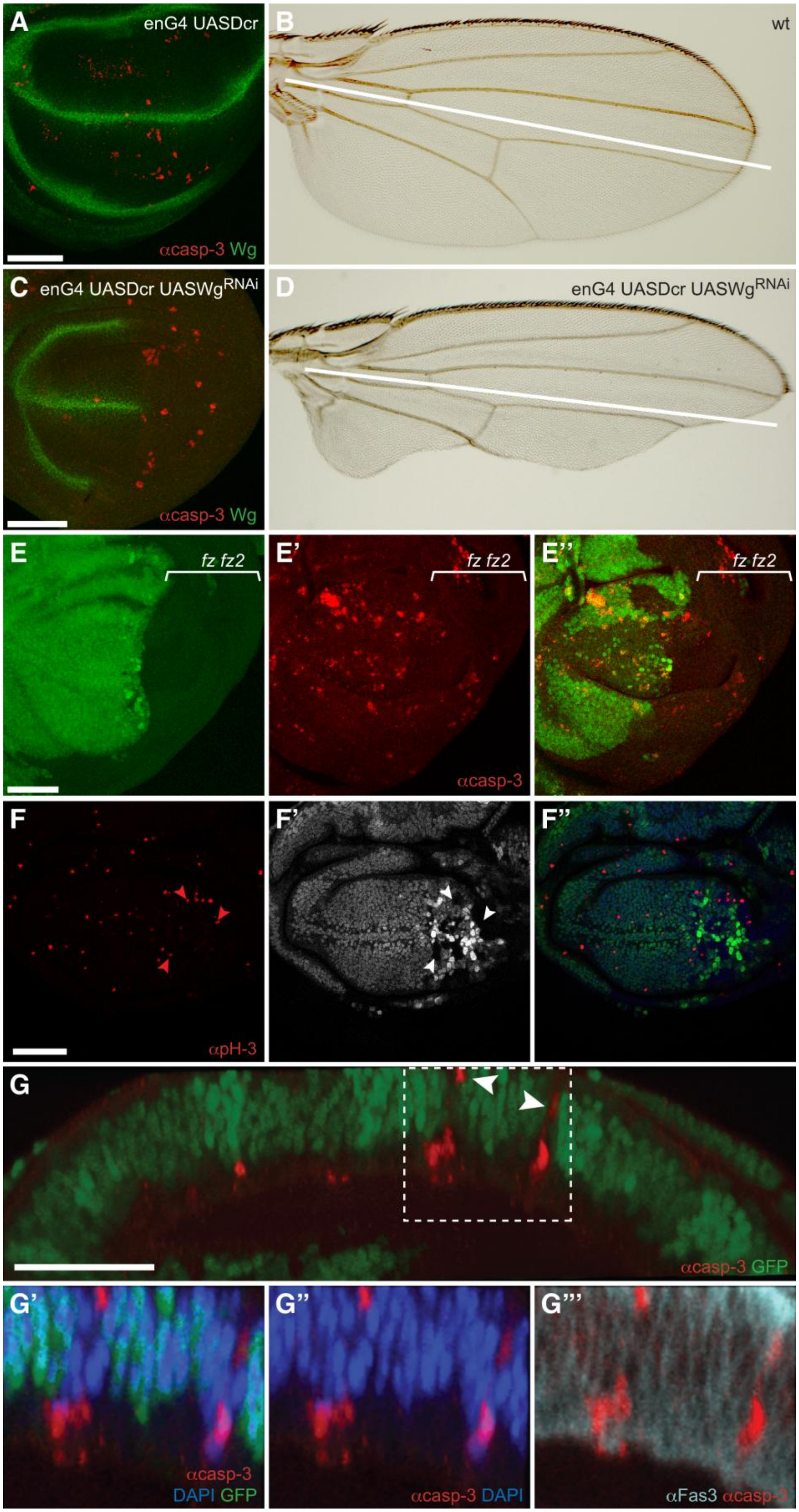

A
enG4 UASDcr
αcasp-3 Wg
B
wt
C
enG4 UASDcr UASWg^RNAi
αcasp-3 Wg
D
enG4 UASDcr UASWg^RNAi
E
fz fz2
E'
fz fz2
αcasp-3
E''
fz fz2
F
αpH-3
F'
F''
G
αcasp-3 GFP
G'
αcasp-3
DAPI GFP
G''
αcasp-3 DAPI
G'''
αFas3 αcasp-3

Figure S1. Survival of Cells Lacking Wg Signalling Depends on Cellular Context
(A) Expression of Dcr-2 in the P compartment (control for panel C) causes increased apoptosis.
(B) Approximate location of the A-P boundary (white line) in a wild type wing.
(C) Coexpression of Dcr-2 and *wg* RNAi in the P compartment does not cause a further increase in anti-Caspase3 staining (by comparison to the situation in control discs, as shown in panel A).
(D) Wing from a larva of the same genotype as in (C). Despite Wg removal, a significant proportion of P cells survives to adulthood and contributes to the wing.
(E-E") Wing disc lacking Fz and Fz2 throughout most of the P compartment (*fzfz2*$^{-/-}$ cells are marked by the absence of GFP in E and E"). No specific increase in the number of caspase-3 positive cells (red in E'-E") is observed in *fz fz2* mutant P compartment (as compared to control wild type P compartments, not shown). Increased cell death in the A compartment is probably due to haploinsufficiency of Minute. Panels (E'-E") show projections of the basal-most sections. All other micrographs show a projection of the whole epithelium.
(F-F") Wing disc in which the P compartment is a mosaic of *cycA* (2XGFP) and *fzfz2* (GFP-negative) mutant cells (same genotype as in Figure 1C). The preparation was stained with anti-Phospho-Histone3 antibody (F-F", red), which marks mitotic cells (arrowheads). Absence of anti-Phospho-Histone3 staining in the *cycA* mutant domain shows that these cells have stopped dividing.
(G-G''') Cross-section of wing imaginal disc harbouring *arrow* mutant clones (GFP-negative). G'-G''' show at higher magnification the area boxed in G. *arrow*$^{-/-}$ cells delaminate from the epithelium and accumulate basally (see also Widmann and Dahmann, 2009). Many of the basally extruded cells are apoptotic, as indicated by caspase-3 staining. In some instances caspase-3 reactivity is observed in *arrow*$^{-/-}$ cells that have not yet delaminated (arrowheads), suggesting that in these cases apoptosis is not a consequence of delamination. Stainings are as indicated (Fas3: Fasciclin3).

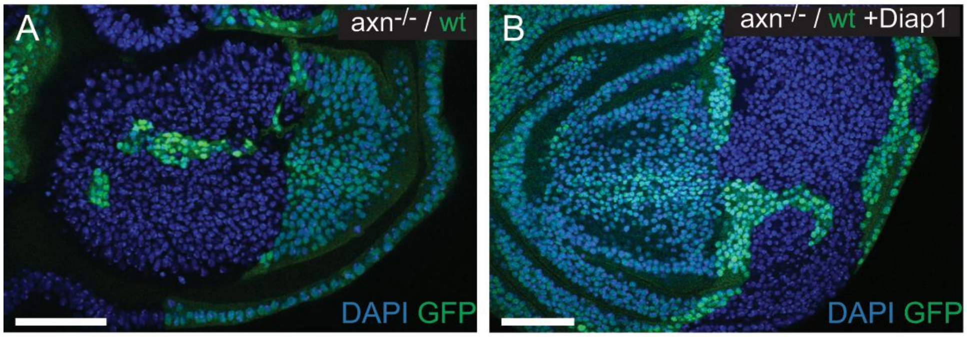

Figure S2. *axn*$^{-/-}$ Cells Outcompete Wild-Type Cells Also in the A Compartment and Expression of DIAP1 Does Not Prevent Axin Mutant Cells from Taking Over a Compartment

(A) *axn*$^{-/-}$ cells (GFP-negative) generated in the anterior compartment outcompete wild-type cells, as they do in the P compartment. Mosaics were generated with *ci*-Gal4 and *UAS-Flp*.
(B) Expression of DIAP1 in the context of an *axin* mosaic P compartment. An equal number of *axin* mutant (GFP-negative) and wild-type cells (GFP-positive) were generated with *engrailed-Gal4* and *UAS-Flp*. At the same time DIAP1 was expressed throughout the P compartment to prevent apoptosis. Under these conditions, wild type cells were still underrepresented (compare to discs in Figures 2D, 3A, 3B), suggesting that differential proliferation rates play also a role in tissue colonization.

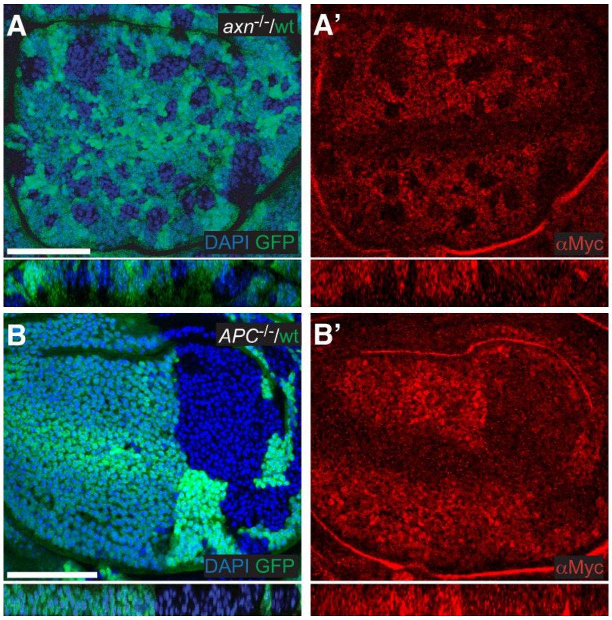

Figure S3. *Axin* and *APC* Mutant Cells Downregulate Myc Expression

(A-B) *axin* mutant cells (GFP negative in A) and *APC* mutant cells (GFP negative in B) display a significant reduction in Myc levels (detected by anti-Myc antibody staining, A', B'). At the bottom of each panel are reconstructions of a cross section through the disc epithelium. Genotypes and staining are as indicated.

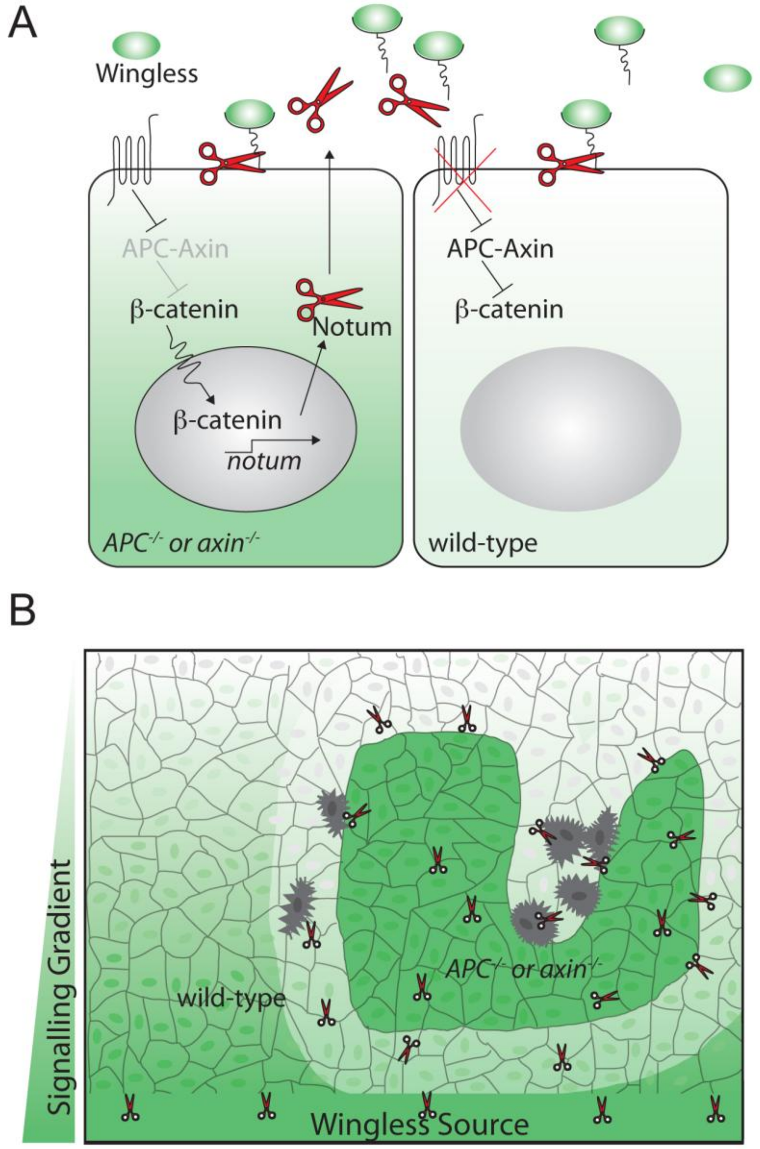

Figure S4. The Role of Notum in Wnt-Induced Cell Competition
Notum is predicted to act as secreted glypican-specific phospholipase (Kreuger et al., 2004; Traister et al., 2007). *notum* expression is activated by Wg signalling and secreted Notum inhibits signal transduction, making Notum a classical secreted feedback inhibitor of the pathway.
(A) By releasing glypicans from the cell surface, Notum (red scissors) reduces the ability of Wg to engage with the signalling receptors. Cells lacking *APC* or *axin*, overactivate the Wg pathway (dark green) irrespectively of whether Wg is present or not. As a result they secrete high levels of Notum, which reduces signalling in surrounding wild type cells (lighter green shading).
(B) A patch of *APC* or *axin* mutant cells express *notum* highly, thus suppressing signalling in surrounding cells. The resulting steep difference in signalling triggers apoptosis (dark grey) in the weaker signalling cells.

Supplemental Experimental Procedures

Drosophila Stocks

The following fly stocks were used*:* *cycA*^{C8LR1} *ubi-GFP FRT2A/TM3* (*cycA* mutation obtained from C. Lehner and recombined for this study with *ubi-GFP FRT2A* obtained from Alex Gould; NIMR, London); *FRT 42D ubi-GFP PCNA*^{775}*/Gla Bc* (I. Salecker; NIMR, London); *y w hs-FLP*; *Sp*/*Gla Bc*; *Dfz1*^{P21}*Dfz2*^{C2} *ri FRT2A/TM6* (G. Struhl; Columbia University, New York); *y w; M(3)i*^{55} *p[nls-GFP]FRT2A/TM6* (F. Schweisguth; Institut Pasteur, Paris); *FRT2A /TM3*(A. Gould); *FRT42D pwn arr*^{2}*/Gla Bc* (G. Struhl); *engrailed-Gal4, UAS-FLP* (J. Casal; University of Cambridge); *FRT82 axin*^{H}*/ TM6* (M. Bienz; MRC-LMB, Cambridge); *FRT82B ubi-GFP* (Bloomington); *w; FRT82B ubi-GFP Rps3/TM6* (Bloomington); *ci-Gal4/CyO* (L.A. Baena-Lopez; NIMR, London); *UAS-FLP* (Bloomington); *UAS-wg*^{RNAi} *UAS-Dcr2/TM6* (Piddini and Vincent, 2009)*; FRT82B APC2*^{g10} *APC*^{Q8}*/TM6* (M.Peifer; University of North Carolina, Chapel Hill); *y w hs-FLP* (Bloomington)*; esg-Gal4, UAS-FLP/CyO* (recombined for this study); *FRT82B* (IBDML; Marseille); *UAS-nt*^{RNAi} (VDRC); *UAS-myc*^{RNAi} (VDRC); *UAS-Dcr-2* (B. Dickson, IMP, Vienna); *UAS-myc* (L.A. Baena-Lopez; NIMR, London); *FRTG13 en-Gal4 arr*^{2} (recombined for this study); *Df3(3L)st-f13 FRT82B axn*^{H}*/TM6* (recombined for this study); *wf*^{141} *FRT82B ubi-GFP/TM3* (recombined for this study); *wf*^{141} *hh-Gal4 UAS-FLP/TM6 TM3* (recombined for this study); *FRTG13 arm-LacZ* (Bloomington).

Genotypes of Discs Displayed in the Main Figures

Figure 1

A, *en-Gal4 UAS-FLP/+ ; FRT2A / ubi-GFP FRT2A*
B, *en-Gal4 UAS-FLP/+*; *Dfz1*^{P21}*Dfz2*^{C2} *ri FRT2A / ubi-GFP FRT2A*
C, *en-Gal4 UAS-FLP/+*; *Dfz1*^{P21}*Dfz2*^{C2} *ri FRT2A / cycA*^{C8LR1} *ubi-GFP FRT2A*
D, *FRT42D arr*^{2}*/FRT 42D ubi-GFP*; *hh-Gal4 UAS-FLP/+*
E-F, *FRT42D arr*^{2}*/FRT 42D ubi-GFP PCNA*^{775}; *hh-Gal4 UAS-FLP/+*

Figure 2

A, *esg-Gal4 UAS-FLP/+; FRT82B/FRT82B ubi-GFP*
B, *esg-Gal4 UAS-FLP/+; FRT82B axn*^{H}*/FRT82B ubi-GFP*
D, *en-Gal4 UAS-FLP/+; FRT82B/FRT82B ubi-GFP*
E and G, *en-Gal4 UAS-FLP/+; FRT82B axn*^{H}*/FRT82B ubi-GFP*
F *en-Gal4 UAS-FLP/+; FRT82B APC2*^{G10} *APC*^{Q8}*/FRT82B ubi-GFP*

Figure 3

A-A", B-B", *en-Gal4 UAS-FLP/+; FRT82B axn*^{H}*/FRT82B ubi-GFP*
C-C", *en-Gal4 UAS-FLP/UAS-myc*^{RNAi}*; FRT82B axn*^{H}*/FRT82B ubi-GFP*
D-D', *en-Gal4 UAS-FLP/UAS-myc; FRT82B axn*^{H}*/FRT82B ubi-GFP*
F, *hs-flp/+; FRT82B axn*^{H}*/FRT82B ubi-GFP*
G, *hs-flp/+; FRT82B/FRT82B ubi-GFP Rps3*
H, *hs-flp/+; FRT82B axn*^{H}*/FRT82B ubi-GFP Rps3*
J, *FRT82B ubi-GFP Rps3/+*
K, *ci-Gal4/UAS-FLP*; *FRT82B axn*^{H}*/ FRT82B ubi-GFP Rps3*

Figure 4
A, E-E' *UAS-Dcr/+; en-Gal4 UAS-FLP/UAS-nt*^{RNAi}*; FRT82B axn*^{H}*/ FRT82B ubi-GFP*
C, *en-Gal4 UAS-FLP/+; FRT82B axn*^{H}*/ FRT82B ubi-GFP*
D, *en-Gal4 UAS-FLP/+; Df3(3L)st-f13 FRT82B axn*^{H}*/ wf*^{141} *FRT82B ubi-GFP*
G-G', *FRTG13 en-Gal4 arr*^{2} */ FRTG13 arm-LacZ; Df3(3L)st-f13/ wf*^{141} *hh-Gal4 UAS-FLP*
H-H', *UAS-Dcr/ hs-flp; en-Gal4 /UAS-nt*^{RNAi}*; M(3)i55 ubi-GFP FRT80/ FRT80*

Genotypes of Discs Displayed in the Supplemental Figures

Figure S1
A, *en-Gal4/+*; *UAS-Dcr-2/ UAS-Dcr-2*
B, *y, w*
C-D, *en-Gal4/+; UAS-Dcr-2/ UAS wg*^{RNAi} *UAS-Dcr-2*
E-E", *en-Gal4 UAS-FLP/+* ; *Dfz1*$^{\text{P21}}$*Dfz2*$^{\text{C2}}$ *ri FRT2A / M(3)i*^{55} *p[nls-GFP]FRT2A*
F-F", *en-Gal4 UAS-FLP/+*; *Dfz1*$^{\text{P21}}$*Dfz2*$^{\text{C2}}$ *ri FRT2A / cycA*$^{\text{C8LR1}}$ *ubi-GFP FRT2A*
G-G'", *hs-FLP/+; FRT42d arr*^{2}*/FRT42D ubi-GFP*

Figure S2
A, *ci-Gal4 UAS-FLP/+*; *FRT82B axn*^{H}*/FRT82B ubi-GFP*
B, *en-Gal4 UAS-FLP/ UAS-DIAP1*; *FRT82B axn*^{H}*/FRT82B ubi-GFP*

Figure S3
A-A', *hs-FLP/+; FRT82B axn*^{H}*/FRT82B ubi-GFP*
B-B', *en-Gal4 UAS-FLP/+; FRT82B APC2* ^{G10} *APC*^{Q8}*/FRT82B ubi-GFP*
